# Comparative Assessment of Sera from Individuals after S-Gene RNA-Based SARS-CoV-2 Vaccination with Spike-Protein-Based and Nucleocapsid-Based Serological Assays

**DOI:** 10.3390/diagnostics11030426

**Published:** 2021-03-03

**Authors:** Anja Dörschug, Hagen Frickmann, Julian Schwanbeck, Elif Yilmaz, Kemal Mese, Andreas Hahn, Uwe Groß, Andreas E. Zautner

**Affiliations:** 1 Institute for Medical Microbiology, University Medical Center Göttingen, 37075 Göttingen, Germany; anja.doerschug@stud.uni-goettingen.de (A.D.); julian.schwanbeck@med.uni-goettingen.de (J.S.); kemal.mese@med.uni-goettingen.de (K.M.); ugross@gwdg.de (U.G.); 2 Institute for Medical Microbiology, Virology and Hygiene, University Medicine Rostock, 18057 Rostock, Germany; hagen.frickmann@med.uni-rostock.de (H.F.); andreas.hahn@uni-rostock.de (A.H.); 3 Department of Microbiology and Hospital Hygiene, Bundeswehr Hospital Hamburg, 20359 Hamburg, Germany; 4 Department of Anesthesiology, University Medical Center Göttingen, 37075 Göttingen, Germany; elif.yilmaz@med.uni-goettingen.de

**Keywords:** SARS-CoV-2, COVID-19, serology, vaccination, surveillance, nucleocapsid, spike protein

## Abstract

Due to the beginning of vaccination against COVID-19, serological discrimination between vaccine-associated humoral response and serology-based surveillance of natural SARS-CoV-2 infections as well as breakthrough infections becomes an issue of relevance. Here, we assessed the differentiated effects of the application of an RNA vaccine using SARS-CoV-2 spike protein epitopes on the results of both anti-spike protein–based serology (EUROIMMUN) and anti-nucleocapsid-based serology (VIROTECH). A total of 80 serum samples from vaccinees acquired at different time points after vaccination was assessed. While positive or borderline serological response in the anti-spike protein assay was observed for all samples (90% both IgG and IgA, 6.3% IgA only, 3.8% borderline IgG only), only a single case of a falsely positive IgM was observed for the anti-nucleocapsid assay as expected due to this assay’s specificity. Positive anti-spike protein antibodies were already detectable in the second week after the first dose of vaccination, with higher titers after the second dose of the vaccine. In conclusion, the combined application of anti-spike protein–based serology and anti-nucleocapsid-based serology will provide a useful option for the discrimination of vaccination response and natural infection.

## 1. Introduction

At the end of 2020, vaccination against COVID-19 was started in Germany with RNA-based vaccines using epitopes of the SARS-CoV-2 spike protein (SARS-2-S) [1] to induce protective immunity [2,3]. Vaccine-induced antibodies against the spike protein can be expected in vaccinees as indicated by the study on safety and immunogenicity prior to authorization [4]. Accordingly, vaccinated individuals without infection by SARS-CoV-2 should develop measurable antibodies in serological assays targeting the SARS-CoV-2 spike protein but not in assays targeting the nucleocapsid (SARS-2-N) protein [5] of SARS-CoV-2.

In the course of the recent months, various studies have assessed the diagnostic performance characteristics of SARS-CoV-2-specific serological assays, indicating imperfect sensitivity and specificity of the assays, with higher reliability of assays targeting IgG compared to IgM- and IgA-specific ones [6,7,8,9,10,11,12,13,14]. Specificity problems with serological assays based on whole viral antigens [15] facilitated the development of more specific assays based on chosen structures of the virus only. Although the viral nucleocapsid as well as the antigens of the viral open reading frames (ORFs) 8 and 3b were shown to elicit the strongest specific antibody responses after SARS-CoV-2-infections [16], serological assays targeting the spike protein and the nucleocapsid protein have been most frequently designed. Serological response against the spike protein has been considered as particularly interesting due to reported interaction with ACE2 (angiotensin-converting enzyme 2) binding [17]. Further, spike-protein-specific antibodies have been reported to persist longer than nucleocapsid-specific antibodies [18], which decrease within weeks to months [19]. Spike proteins offer even better sensitivity if the spike protein is applied as trimer and not as monomer in the serological assay [20]. Indeed, the trimeric spike protein was one of the first implemented as the primary antigen in various serological assays [21]. Intensity of humoral immunoreactivity toward the SARS-CoV-2 spike protein was shown to be influenced by disease severity and smoking status [22].

In spite of the shorter half-life of nucleocapsid-specific antibodies [18,19], their combination with antibodies against the receptor-binding domain of the spike protein [23] as well as their assessment alone [24] were associated with excellent specificity. In the short-term range of 2 weeks after infection, there are no differences between sensitivity of assays targeting antibodies against the nucleocapsid or the spike protein [25]. In recent comparisons of nucleocapsid- and spike-protein-based assays, no unambiguous superiority of one approach or the other could be identified [26,27,28,29]. In early infection states, sensitivity of anti-nucleocapsid antibodies can even be higher than that of anti-spike protein antibodies [24,30,31]. Other studies suggested a higher degree of inconsistency of the abundance of anti-nucleocapsid antibodies compared to anti-spike protein antibodies after SARS-CoV-2-infections [32,33]. Furthermore, spike-protein-based assays were reported to better predict the outcome of neutralization assays [34].

Considering the reported good specificity results of nucleocapsid assays [23,24], vaccination with SARS-CoV-2 spike-protein-based vaccines should elicit robust anti-spike protein antibodies as its humoral response but ought to show no cross-reaction with anti-nucleocapsid-antibody-specific assays. It should therefore be possible to differentiate between a vaccination response and at least recent natural infections with SARS-CoV-2 [18,19] on the basis of these different test strategies.

In this study, a serological assay by EUROIMMUN targeting the spike protein and an assay by VIROTECH targeting the nucleocapsid protein of SARS-CoV-2 were comparatively applied for the testing of volunteers after mRNA-based vaccination with mRNA encoding the spike protein as described [1,2,3,4]. Both the EUROIMMUN and the VIROTECH assay had already been evaluated in previous assessments [12,35,36]. By doing so, it was intended to confirm the expected differentiated response with reactivity in the spike-protein-based assay, but without reactivity in the nucleocapsid-based assay in vaccinated individuals without previous SARS-CoV-2 infection.

## 2. Materials and Methods

### 2.1. Sample Collection

Assessed samples comprised 80 sera of volunteers vaccinated with the Comirnaty COVID-19 mRNA vaccine from BioNTech (Mainz, Germany) and Pfizer (Puurs, Belgium) [36], collected after the first or second vaccination. The sera were stored in the refrigerator at 2 to 8 °C prior to assessment for a maximum of 2 days. The vaccinees were healthcare workers at the University Medical Center Göttingen, Germany.

### 2.2. Serological Assays

The compared serological assays comprised the spike-protein-based EUROIMMUN COVID-19 IgG/IgA assay (EUROIMMUN, Lübeck, Germany; referred to as “EUROIMMUN assay” in the following) and the nucleocapsid-based VIROTECH SARS-CoV-2 IgA/IgM/IgG ELISA (Rüsselsheim am Main, Germany; referred to as “VIROTECH assay” in the following). Both assays were performed exactly as described by the manufacturers.

### 2.3. Statistics

Correlation between the titers of the anti-spike protein antibodies and the time from first and second vaccination until sample acquisition as well as the vaccinees’ age at sample acquisition was calculated applying Spearman’s correlation coefficient after conducting a significant Shapiro–Wilk test for normal distribution.

Wilcoxon’s rank sum test was applied to detect statistical differences for the anti-spike protein antibody titers between males and females and between individuals who received one or two vaccinations.

### 2.4. Ethics

The study was ethically approved by the institutional ethics board of the University Medical Center Göttingen (identification code 21/05/20, provided on 21 May 2020).

## 3. Results

### 3.1. Vaccinees

The assessed 80 vaccinees comprised 36 (45.0%) males and 44 (55.0%) females. At the time of sample acquisition, the mean (± standard deviation SD) age in years was 39.4 (± 1.4), and the median age (interquartile range) was 37 (29, 49). At total of 53/80 (66.3%) had received the first and the second vaccine, 27/80 (33.8%) only the first vaccine. For the patients who had only received the first vaccine, the mean (± SD) number of days after this vaccine until sample acquisition was 16.5 (± 5.4), for the patients who had received both vaccines it was 4.5 (± 4.6). The raw study data on the vaccinees are shown in the Appendix A, Table A1.

### 3.2. Serological Test Results

In the spike-protein-specific EUROIMMUN assays, all assessed 80 vaccinees showed at least one positive (*n* = 77/80, 96.3%) or borderline (*n* = 3/80, 3.8%) result for either the immunoglobulin subclass A, G, or both. In detail, in the EUROIMMUN IgG assay 72/80 (90.0%) positive and 3/80 (3.8%) borderline results were observed. In the EUROIMMUN IgA assay, 77/80 (96.3%) showed positive results. Of note, the three cases of borderline EUROIMMUN IgG results were associated with the three negative EUROIMMUN IgA results without exemption, while positive IgA results were always associated with positive IgG results.

The three cases with negative EUROIMMUN IgA and borderline EUROIMMUN IgG comprised a 48-year-old female 19 days after her first vaccine, a 61-year-old female 1 day after her second vaccine, and a 60-year-old male 3 days after his second vaccine, respectively. The five cases with positive EUROIMMUN IgA result but negative EUROIMMUN IgG result consisted of a 21-, 51-, and a 63-year-old female and a 50-year-old male 11 days after their first vaccine, as well as a 23-year-old male 9 days after his first vaccine, respectively.

No positive results were observed for the immunoglobulin subclasses IgG and IgA of the nucleocapsid-specific VIROTECH assay. For IgM, a positive VIROTECH signal was recorded in a 51-year-old female 3 days after her second vaccine.

The raw data are provided in the Appendix A, Table A2.

### 3.3. Correlation of the Time in Days between the Last Vaccine and Sample Acquisition, Age, Sex and the Measured Titers

Spearman’s correlation coefficient between the time from the first and second vaccination and the titers of the anti-spike protein antibodies are indicated in Table 1. As shown there, respective correlation was weak for IgA, especially in patients who had received only one vaccine at the time of sample acquisition. It was slightly better after two vaccines, in spite of a tendency for higher titers after longer time intervals. For IgG, the correlation was better for all assessed situations.

Table 2 indicates Spearman’s correlation coefficients between the age at the sample acquisition date and the anti-spike protein antibody titers. A non-significant trend for higher titers in young age groups was observed for both IgA and IgG antibodies.

The anti-spike protein antibody titers among males and females were compared by conducting Wilcoxon’s rank sum test and were not statistically significant assuming a level of significance of 0.05 (Table 3).

**Figure 1:**
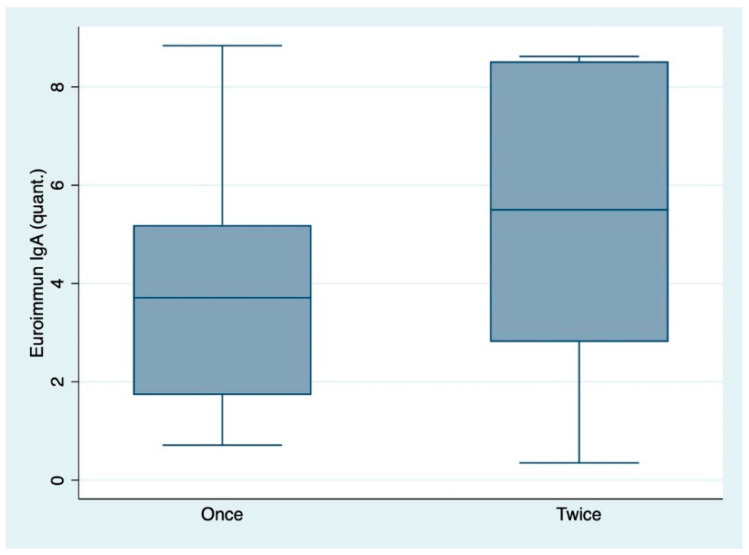
Box-plots indicating the difference of IgA antibody titers (as ratio of the patient sample over the extinction of the calibrator) among individuals who received one or two vaccinations. “quant.”: quantitative IgA titers. Once: one vaccination only. Twice: two vaccinations.

**Figure 2:**
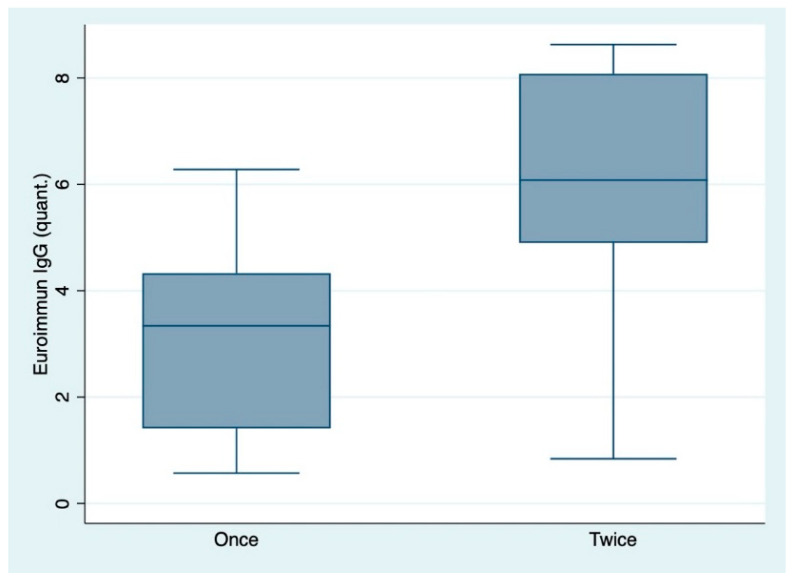
Box-plots indicating the difference of IgG antibody titers (as ratio of the patient sample over the extinction of the calibrator) among individuals who received one or two vaccinations. “quant.”: quantitative IgG titers. Once: one vaccination only. Twice: two vaccinations.

The anti-spike protein antibody titers among individuals who received one or two vaccinations were tested by conducting Wilcoxon’s rank sum test and were statistically significant assuming a level of significance of 0.05 for both subclasses IgA and IgG. The titers were higher among individuals who received both vaccinations (Table 4, Figure 1 and Figure 2).

## 4. Discussion

The study was conducted to differentiate the vaccination with RNA-based vaccines using epitopes of the SARS-CoV-2 spike protein (SARS-2-S) [1,2,3,4] as a proof-of-principle assessment. First of all, positive or borderline results for anti-spike protein antibodies were observed for all assessed vaccinees, irrespective of vaccination status with just one or with two vaccine doses. In contrast, anti-nucleocapsid IgA and IgG antibodies did not occur in the vaccinees. The single positive IgM result among the 80 assessed samples is as expected in line with the previously calculated specificity of the applied VIROTECH anti-nucleocapsid-IgM assay between 94% and 96.7% as indicated in a previous study [12]. Accordingly, the reported single positive IgM result can most likely be judged as a false positive. Hypothetically, a very early stage of a breakthrough infection is an alternative explanation, so follow-up assessment is recommended in case of such a serological reaction pattern. In this specific case, serological follow-up was unfeasible as the respective volunteer agreed to one blood sample acquisition only. However, in retrospect, when asked, the subject reported no symptoms of a mild upper respiratory tract infection at the relevant time.

Considering the acceptable sensitivity and specificity of the VIROTECH assay with samples from patients with COVID-19 as shown elsewhere [12], our data suggest that a combination of anti-spike protein–based serology and anti-nucleocapsid-based serology can be used to discriminate spike-protein-based vaccination-induced antibody titers from antibodies after acute SARS-CoV-2 infections as well as from potential antibodies due to breakthrough infections in spite of vaccination.

Secondly, it could be shown that anti-spike protein antibodies are already measurable early after vaccination. While 72 vaccinees showed a combination of positive IgG and IgA against the SARS-CoV-2 spike protein, five individuals with samples acquired at very early time points—9 to 11 days after the first vaccine—expressed IgA but still no IgG antibodies. This pattern was not observed at later time points of sample acquisition, suggesting its occurrence at early stages after vaccination only.

A pattern of borderline IgG anti-spike protein antibodies without measurable IgA antibodies, which was observed, compared to the study median, in older vaccinees once after the first and twice after the second vaccine dose, calls for further follow-up. Although this pattern was observed in less than 5% of the vaccinees, it raises the question of potentially insufficient humoral response toward the vaccination applied.

Thirdly, associations of antibody-titers with factors such as age, sex, and time between vaccination and serum sample acquisition were assessed. While respective correlations were generally weak, at least significance for higher anti-spike protein antibody titers in individuals after the second vaccination compared to patients with only one vaccine dose could be shown.

The study has a number of limitations. Firstly, the comparable low number of only 80 vaccinees allows a preliminary interpretation only. Secondly, the intervals between vaccination and sample acquisition were arbitrary and defined by the accessibility of the vaccinees for the sample acquisition. Thirdly, a sample acquisition on a daily basis would have been desirable to assess the seroconversion process. However, this procedure was not feasible for organizational reasons. Fourthly, neutralizing potential of the measured antibodies was not tested, as the correlation of the results of the used serological assays to the results from neutralization tests has already been demonstrated in preliminary studies [7]. Also, the scope of the work was not the confirmation of the neutralizing potential of vaccine-related antibodies but just the establishing of a ready-to-perform approach of serological discrimination of post-vaccination antibodies and wildtype virus infection–related ones in the diagnostic routine.

## 5. Conclusions

In a small group of recently vaccinated individuals, anti-spike protein antibodies were very consistently recorded, while anti-nucleocapsid antibodies were virtually absent. In spite of the limitations mentioned, the assessment strongly suggests combined application of anti-spike protein–based serology and anti-nucleocapsid-based serology for the discrimination of responses after spike-protein-based vaccination and natural infections. Although not directly assessed in this study, it is likely that this may also apply to breakthrough infections. Positive anti-spike protein antibody results can be expected early after vaccination, although higher titers are seen after the second vaccine. Larger studies are recommended to confirm these preliminary results. From the practical clinical point of view, a combination of serological assays for the discrimination of anti-spike protein antibodies and anti-nucleocapsid antibodies is recommended, if a recent breakthrough infection with SARS-CoV-2 is to be confirmed in vaccinees for clinical, hygiene-related, or surveillance purposes.

## Figures and Tables

**Table 1:** Spearman’s correlation coefficient between the time from the first and second vaccination and the titers of the anti-spike protein antibodies. The *p*-values for the titers of the anti-spike protein antibodies and the time from first and second vaccination were 0.00003 and <0.0001, respectively.

Population	*n*	Time from Vaccination		Anti-Spike Protein Antibodies		Spearman’s Correlation Coefficient with 0.95 Confidence Interval
		Mean (SD)	Median (q25, q75)	Mean (SD)	Median (q25, q75)	
IgA ^1^: All individuals	80	22.53 (5.85)	22.50 (21, 27)	4.88 (2.73)	4.94 (2.48, 8.03)	0.479 [0.274, 0.676].
IgA ^1^: Individuals with only first vaccination	27	16.48 (5.38)	19 (11, 21)	3.87 (2.28)	3.71 (1.73, 5.19)	0.0075 [−0.342, 0.357
IgA ^2^: Individuals with first and second vaccination	53	4.49 (4.57)	3 (1, 7)	5.39 (2.82)	5.5 (2.81, 8.52)	0.539 [0.261, 0.760]
IgG ^1^: All individuals	80	22.53 (5.85)	22.50 (21, 27)	4.96 (2.47)	4.97 (3.02, 7.52)	0.787 [0.646, 0.858]
IgG ^1^: Individuals with only first vaccination	27	16.48 (5.38)	19 (11, 21)	2.96 (1.69)	3.34 (1.41, 4.33)	0.638 [0.384, 0.810]
IgG ^2^: Individuals with first and second vaccination	53	4.49 (4.57)	3 (1, 7)	5.98 (2.18)	6.08 (4.90, 8.08)	0.698 [0.468, 0.839]

^1^ Time from first vaccination, ^2^ time from second vaccination.

**Table 2:** Spearman’s correlation coefficients between the age at the sample acquisition date and the anti-spike protein antibody titers. The *p*-value for age at sample acquisition was 0.00238. The *p*-value for anti-spike protein IgA antibodies was 0.00008 and for IgG anti-bodies 0.00108.

Population	*n*	Age in Years at Sample Acquisition		Anti-Spike Protein Antibodies		Spearman’s Correlation Coefficient with 0.95 Confidence Interval
		Mean (SD)	Median (q25, q75)	Mean (SD)	Median (q25, q75)	
IgA ^1^: All individuals	80	39.4 (12.31)	37 (29, 49)	4.88 (2.73)	4.94 (2.48, 8.03)	−0.153 [−0.382, 0.092]
IgA ^1^: Individuals with only first vaccination	27	40.25 (13.69)	39 (28, 50)	3.87 (2.28)	3.71 (1.73, 5.19)	−0.216 [−0.599, 0.187]
IgA ^2^: Individuals with first and second vaccination	53	38.96 (11.66)	36 (30, 48)	5.39 (2.82)	5.5 (2.81, 8.52)	−0.078 [−0.367, 0.207]
IgG ^1^: All individuals	80	39.4 (12.31)	37 (29, 49)	4.96 (2.47)	4.97 (3.02, 7.52)	−0.213 [−0.440, 0.044]
IgG ^1^: Individuals with only first vaccination	27	40.25 (13.69)	39 (28, 50)	2.96 (1.69	3.34 (1.41, 4.33)	−0.142 [−0.569, 0.296]
IgG ^2^: Individuals with first and second vaccination	53	38.96 (11.66)	36 (30, 48)	5.98 (2.18)	6.08 (4.90, 8.08)	−0.211 [−0.501, 0.035]

^1^ Time from first vaccination, ^2^ time from second vaccination.

**Table 3:** Comparison of spike protein antibody titers among males and females applying Wilcoxon’s rank sum test.

Population	Males			Females			Wilcoxon Rank Sum Test, *p*-Value
	*n*	Mean (SD)	Median (q25, q75)	*n*	Mean (SD)	Median (q25, q75)	
IgA ^1^: All individuals	36	5.39 (2.63)	5.30 (2.91, 8.42)	44	4.46 (2.77)	4.00 (1.77, 7.10)	0.1255
IgA ^1^: Individuals with only first vaccination	12	4.59 (2.26)	5.00 (2.86, 5.68)	15	3.29 (2.19)	3.25 (1.25, 5.19)	0.1959
IgA ^2^: Individuals with first and second vaccination	24	5.78 (2.76)	6.23 (2.91, 8.52)	29	5.06 (2.87)	4.90 (2.56, 8.52)	0.4017
IgG ^1^: All individuals	36	4.87 (2.35)	4.92 (2.92, 6.48)	44	5.03 (2.60)	5.00 (3.16, 7.69)	0.7866
IgG ^1^: Individuals with only first vaccination	12	3.13 (1.69)	3.07 (2.13, 4.33)	15	2.83 (1.74)	3.45 (1.03, 3.96)	0.6256
IgG ^2^: Individuals with first and second vaccination	24	5.74 (2.16)	5.72 (4.46, 8.09)	29	6.17 (2.22)	6.67 (4.93, 8.08)	0.4529

^1^ Time from first vaccination, ^2^ time from second vaccination.

**Table 4:** Comparison of spike protein antibody titers among individuals who received one or two vaccinations applying Wilcoxon’s rank sum test.

Population	Only First Vaccination			First and Second Vaccination			Wilcoxon Rank Sum Test, *p*-Value
	*n*	Mean (SD)	Median (q25, q75)	*n*	Mean (SD)	Median (q25, q75)	
IgA: All individuals	27	3.87 (2.28)	3.71 (1.73, 5.19)	53	5.39 (2.82)	5.50 (2.81, 8.52)	0.0217
IgG: All individuals	27	2.96 (1.69)	3.34 (1.41, 4.33)	53	5.98 (2.18)	6.08 (4.90, 8.08)	<0.0001

## Data Availability

All relevant data are provided in the manuscript and its tables.

## Appendix A

**Table A1:** Vaccinee-specific raw data.

Study ID	Sex (1 = Male, 2 = Female)	Age at Sample Acquisition (Years)	Date of First Vaccine	Date of Second Vaccine	Date of sAmple Acquisition
1	1	19	2021-01-21		2021-02-01
2	2	21	2021-01-21		2021-02-01
3	2	25	2021-01-21		2021-02-01
4	2	36	2021-01-21		2021-02-01
5	2	51	2021-01-21		2021-02-01
6	1	50	2021-01-21		2021-02-01
7	2	63	2021-01-21		2021-02-01
8	2	49	2021-01-12		2021-02-02
9	1	62	2021-01-15		2021-02-02
10	2	48	2021-01-20		2021-02-02
11	1	24	2021-01-14		2021-02-02
12	2	30	2021-01-12		2021-02-02
13	2	48	2021-01-14		2021-02-02
14	1	44	2021-01-13		2021-02-02
15	1	54	2021-01-11	2021-02-01	2021-02-02
16	1	31	2021-01-11	2021-02-01	2021-02-02
17	2	28	2021-01-11	2021-02-01	2021-02-02
18	1	31	2021-01-11		2021-02-02
19	1	48	2021-01-11	2021-02-01	2021-02-02
20	2	61	2021-01-11	2021-02-01	2021-02-02
21	1	32	2021-01-12		2021-02-02
22	2	45	2021-01-11		2021-02-02
23	1	34	2021-01-12		2021-02-02
24	1	33	2021-01-11		2021-02-02
25	2	32	2021-01-11	2021-02-01	2021-02-02
26	1	40	2021-01-11	2021-02-01	2021-02-03
27	1	26	2021-01-12	2021-02-02	2021-02-03
28	1	41	2021-01-07	2021-02-01	2021-02-03
29	2	27	2021-01-13		2021-02-03
30	1	43	2021-01-12	2021-02-02	2021-02-03
31	2	48	2021-01-08	2021-02-01	2021-02-04
32	2	46	2021-01-13	2021-01-03	2021-02-04
33	1	34	2021-01-11	2021-02-01	2021-02-04
34	2	50	2021-01-12	2021-02-02	2021-02-04
35	2	49	2021-01-13		2021-02-04
36	2	28	2021-01-11	2021-02-01	2021-02-04
37	1	60	2021-01-11	2021-02-01	2021-02-04
38	2	28	2021-01-18		2021-02-04
39	2	34	2021-01-11	2021-02-01	2021-02-04
40	2	54	2021-01-14		2021-02-04
41	1	60	2021-01-20		2021-02-04
42	1	39	2021-02-01		2021-02-04
43	2	25	2021-01-11	2021-02-01	2021-02-04
44	1	23	2021-01-26		2021-02-04
45	2	62	2021-01-15		2021-02-05
46	2	25	2021-01-12	2021-02-02	2021-02-05
47	1	48	2021-01-14	2021-02-04	2021-02-05
48	1	42	2021-01-12	2021-02-02	2021-02-05
49	2	30	2021-01-14	2021-02-04	2021-02-05
50	2	41	2021-01-12	2021-02-02	2021-02-05
51	1	23	2021-01-12	2021-02-02	2021-02-05
52	2	54	2021-01-11	2021-02-04	2021-02-05
53	2	34	2021-01-12	2021-02-02	2021-02-05
54	2	39	2021-01-08	2021-02-01	2021-02-06
55	2	53	2021-01-11	2021-02-01	2021-02-08
56	2	50	2021-01-08	2021-02-01	2021-02-08
57	2	64	2021-01-08	2021-02-01	2021-02-08
58	2	38	2021-01-11	2021-02-01	2021-02-08
59	1	43	2021-01-12	2021-02-02	2021-02-08
60	2	36	2021-01-07	2021-02-01	2021-02-08
61	2	63	2021-01-11	2021-02-01	2021-02-08
62	1	42	2021-01-11	2021-02-01	2021-02-08
63	1	39	2021-01-08	2021-02-01	2021-02-08
64	2	51	2021-01-15	2021-02-05	2021-02-08
65	1	33	2021-01-12	2021-02-02	2021-02-08
66	2	31	2021-01-13	2021-02-03	2021-02-09
67	1	33	2021-01-11	2021-02-01	2021-02-09
68	1	32	2021-01-18	2021-02-08	2021-02-09
69	1	30	2021-01-11	2021-02-01	2021-02-09
70	2	58	2021-01-12	2021-02-02	2021-02-09
71	1	28	2021-01-18	2021-02-08	2021-02-09
72	2	28	2021-01-13	2021-02-03	2021-02-09
73	2	33	2021-01-12	2021-02-04	2021-02-09
74	1	31	2021-01-12	2021-02-04	2021-02-09
75	2	27	2021-01-13	2021-02-03	2021-02-09
76	1	25	2021-01-11	2021-02-01	2021-02-09
77	1	23	2021-01-12	2021-02-02	2021-02-09
78	2	57	2021-01-12	2021-02-04	2021-02-09
79	1	25	2021-01-12	2021-02-08	2021-02-09
80	2	27	2021-01-13	2021-02-03	2021-02-09

**Table A2:** Raw data of the serological test results—for the VIROTECH results, only qualitative data are shown.

Study ID	Euroimmun IgA (Qualitative, 0 = Negative, 1 = Positive)	Euroimmun IgG (Qualitative, 0 = Negative, 1 = posItive, 3 = Borderline)	Euroimmun IgA (Ratio of the Patient Sample over the Extinction of the Calibrator)	Euroimmun IgG (Ratio of the Patient Sample Over the Extinction of the Calibrator)	Virotech IgA (Qualitative, 0 = Negative, 1 = Positive)	Virotech IgM (Qualitative, 0 = Negative, 1 = Positive)	Virotech IgG (Qualitative, 0 = Negative, 1 = Positive)
1	1	1	8.84	1.41	0	0	0
2	1	0	1.73	0.57	0	0	0
3	1	1	4.1	2.20	0	0	0
4	1	1	5.2	3.62	0	0	0
5	1	0	3.25	0.63	0	0	0
6	1	0	1.99	0.64	0	0	0
7	1	0	1.15	0.62	0	0	0
8	1	1	7.86	4.44	0	0	0
9	1	1	7.41	4.26	0	0	0
10	1	1	1.32	1.46	0	0	0
11	1	1	3.71	4.37	0	0	0
12	1	1	5.19	5.54	0	0	0
13	0	3	0.71	1.03	0	0	0
14	3	1	0.95	3.34	0	0	0
15	1	1	1.89	3.03	0	0	0
16	1	1	6.26	5.47	0	0	0
17	1	1	1.82	5.02	0	0	0
18	1	1	6.18	4.41	0	0	0
19	1	1	1.76	2.48	0	0	0
20	0	3	0.35	0.84	0	0	0
21	1	1	3.24	4.33	0	0	0
22	1	1	1.25	3.94	0	0	0
23	1	1	4.94	6.28	0	0	0
24	1	1	5.07	2.13	0	0	0
25	1	1	3.67	4.93	0	0	0
26	1	1	5.41	5.24	0	0	0
27	1	1	3.02	4.90	0	0	0
28	1	1	4.94	5.99	0	0	0
29	1	1	1.11	5.44	0	0	0
30	1	1	2.55	4.95	0	0	0
31	1	1	3.96	5.62	0	0	0
32	1	1	1.61	2.68	0	0	0
33	1	1	2.48	3.68	0	0	0
34	1	1	3.32	3.02	0	0	0
35	1	1	4.05	3.96	0	0	0
36	1	1	2.26	4.99	0	0	0
37	0	3	0.56	1.09	0	0	0
38	1	1	6.57	3.86	0	0	0
39	1	1	4.9	4.47	0	0	0
40	1	1	2.33	1.77	0	0	0
41	1	1	5.16	2.77	0	0	0
42	1	1	2.48	2.81	0	0	0
43	1	1	6.44	6.67	0	0	0
44	1	0	5.19	0.85	0	0	0
45	1	1	3.55	3.45	0	0	0
46	1	1	3.31	6.17	0	0	0
47	1	1	8.62	5.63	0	0	0
48	1	1	5.51	2.40	0	0	0
49	1	1	4.16	7.57	0	0	0
50	1	1	6.96	6.08	0	0	0
51	1	1	2.81	5.37	0	0	0
52	1	1	2.56	2.00	0	0	0
53	1	1	1.14	4.32	0	0	0
54	1	1	3.13	7.47	0	0	0
55	1	1	5.15	7.82	0	0	0
56	1	1	8.59	8.00	0	0	0
57	1	1	8.52	8.50	0	0	0
58	1	1	8.52	8.49	0	0	0
59	1	1	8.52	8.12	0	0	0
60	1	1	8.52	8.15	0	0	0
61	1	1	8.52	7.88	0	0	0
62	1	1	8.32	8.06	0	0	0
63	1	1	8.52	8.26	0	0	0
64	1	1	1.73	3.31	0	1	0
65	1	1	8.52	8.23	0	0	0
66	1	1	8.33	8.63	0	0	0
67	1	1	8.52	8.24	0	0	0
68	1	1	8.21	5.96	0	0	0
69	1	1	8.52	8.13	0	0	0
70	1	1	8.52	8.30	0	0	0
71	1	1	3.81	4.03	0	0	0
72	1	1	8.52	8.58	0	0	0
73	1	1	7.25	8.08	0	0	0
74	1	1	6.2	6.69	0	0	0
75	1	1	1.21	6.80	0	0	0
76	1	1	8.52	8.23	0	0	0
77	1	1	8.52	7.99	0	0	0
78	1	1	5.5	6.50	0	0	0
79	1	1	6.95	5.81	0	0	0
80	1	1	8.52	8.08	0	0	0
